# Novel Targeted Agents in Advanced and Recurrent Low-Grade Serous Ovarian Cancer: A Silver Lining in the Therapy of a Chemoresistant Disease?

**DOI:** 10.3390/cancers16193268

**Published:** 2024-09-26

**Authors:** Arina Onoprienko, Thomas Bartl, Christoph Grimm, Nicole Concin, Stephan Polterauer

**Affiliations:** Department of Obstetrics and Gynecology, Division of General Gynecology and Gynecologic Oncology, Medical University of Vienna, 1090 Vienna, Austria; arina.onoprienko@meduniwien.ac.at (A.O.); christoph.grimm@meduniwien.ac.at (C.G.); nicole.concin@meduniwien.ac.at (N.C.); stephan.polterauer@meduniwien.ac.at (S.P.)

**Keywords:** ovarian neoplasms, rare diseases, molecular targeted therapy, endocrine treatment, letrozole, trametinib

## Abstract

**Simple Summary:**

Low-grade serous ovarian cancer is a rare and complex subtype that usually develops slowly and predominantly affects younger women. Conventional treatment, which generally involves a combination of primary surgery and chemotherapy, often fails, leading to frequent recurrences. Due to the rarity of this cancer subtype, there are limited clinical data available to guide optimal treatment decisions. However, recent research is focusing on new, promising therapies, including targeted treatments that address specific molecular pathways and hormonal therapies. These emerging options offer hope for improved outcomes by enabling more personalized and effective management. This review aims to address the current treatment challenges and summarize the innovative therapies and their potential to enhance treatment strategies, aiming to provide better management for patients with this uncommon ovarian cancer subtype.

**Abstract:**

Low-grade serous ovarian carcinoma (LGSOC) is a rare subtype of epithelial ovarian cancer, characterized by a unique molecular background and specific clinical behavior. A growing body of molecular data underscores LGSOC as a distinct disease entity; however, clinical evidence on the optimal treatment regimens for LGSOC remains limited due to the low incidence of the disease. Consequently, treatment recommendations for LGSOC are still often derived from findings on the more common high-grade serous ovarian carcinoma (HGSOC) and typically focus on radical cytoreductive surgery and platinum-based chemotherapy. Since LGSOCs typically exhibit only limited responsiveness to platinum-based chemotherapy, the clinical management of advanced and recurrent LGSOCs remains a significant therapeutic challenge and often results in limited treatment options and suboptimal outcomes. Recent advances in molecular profiling and the identification of new, promising targets, such as the mitogen-activated protein kinase (MAPK) pathway, offer hope for improving both the prognosis and health-related quality of life in affected patients. Given the high unmet clinical need to establish new therapeutic standards beyond cytotoxic chemotherapy, this review aims to summarize the most promising molecular targets and emerging targeted agents.

## 1. Introduction

Low-grade serous ovarian cancer (LGSOC) is a rare subtype of epithelial ovarian cancer, with distinct histopathologic features, molecular background, and clinical behavior [1]. LGSOCs account for less than 10% of all epithelial ovarian cancers in the Western world and is typically associated with a younger age at primary diagnosis and slower tumor growth but with relative resistance to platinum-based chemotherapy regimens as compared to the more prevalent high-grade serous ovarian cancer (HGSOC) [2,3,4]. Histologically, LGSOC is characterized by a milder degree of nuclear atypia and lower proliferative activity [5]. Whereas HGSOC is assumed to originate from malignant transformations of the tubal epithelium and is strongly associated with both *TP53* driver mutations and homologous recombination repair pathway aberrations, LGSOCs frequently emerge as localized lesions from serous borderline tumors, characterized by hormone receptor positivity and frequently driven by mutations in the mitogen-activated protein kinase (MAPK) pathway (Table 1) [6,7,8,9,10]. Clinically, its pathognomonic lower proliferative activity is a double-edged sword; as compared to HGSOC, its slower growth favors earlier diagnosis and contributes to a longer overall survival both in early and advanced stages [11]. However, it also results in a relative resistance to platinum-based chemotherapy regimens, with response rates ranging between 4.0 and 23.1% to first-line therapy, 2.1 and 4.9% to platinum re-challenge, and 9.1 and 15.5% to paclitaxel mono-chemotherapy. However, it is to be noted, that response rate data are either derived from retrospective analyses or from very small subgroup analyses from larger trials [4,12]. Even though LGSOC was endorsed as a distinct disease entity by Shih and Kurman back in 2004, its low incidence has been hampering both specific translational and clinical research ever since [13]. Clinical evidence on therapeutic strategies is therefore traditionally derived from clinically and molecularly different HGSOCs, as earlier studies often did either not differentiate serous ovarian carcinoma or included both LGSOCs and HGSOCs in therapeutic studies. Therefore, radical primary cytoreductive surgery followed by six cycles of adjuvant carboplatin and paclitaxel remains the backbone of primary therapy [14,15]. Given the inherently low sensitivity to chemotherapy, adjuvant platinum-based chemotherapy likely offers limited prognostic benefit, while posing considerable toxicity risks. Outweighing the therapeutic efficacy of cytotoxic chemotherapy against its toxicity is therefore becoming increasingly relevant following disease recurrence [12]. The present review aims to highlight the urgent unmet clinical need for more personalized treatment strategies to improve therapeutic efficacy while avoiding unnecessary toxicity and to provide an overview of the most promising emerging therapeutic options to achieve this goal.

## 2. First-Line Treatment Strategies

Upon diagnosis, a referral to a specialized gynecologic oncology center is to be considered, as the adherence to specified quality indicators has been demonstrated to ensure that patients receive the appropriate state-of-the-art therapy and treatment in specialized hospitals, which has been associated with improved outcomes [21,22,23].

Analogous to HGSOCs, primary cytoreductive surgery, usually followed by six cycles of platinum-based chemotherapy, remains the cornerstone of primary LGSOC treatment. With LGSOCs being typically less sensitive to conventional chemotherapy, and therapeutic alternatives still being under investigation, successful primary cytoreduction holds a relatively greater significance as compared to HGSOCs. Neoadjuvant approaches should therefore be avoided, if possible [14]. The absence of macroscopic residual disease following a primary debulking surgery remains the most important prognostic factor of first-line therapy [5]. While evidence on the efficacy of adjuvant platinum-based chemotherapy +/− bevacizumab is limited, it remains an integral part of the ESGO–ESMO–ESP (European Society for Medical Oncology, European Society of Gynecological Oncology, European Society of Pathology) and NCCN (National Comprehensive Cancer Network) guidelines [14,15]. Given the high rates of estrogen receptor expression in LGSOCs, antihormonal therapy can be considered a cost-effective option for maintenance treatment with relatively limited toxicity [14,17]. Encouraging response rates and potential survival benefits have been reported; however, evidence remains limited to retrospective studies [24].

### 2.1. Primary Cytoreductive Surgery

As achieving no residual macroscopic disease during primary surgery has emerged as the strongest prognostic factor across multiple studies, upfront cytoreductive surgery with the goal of achieving no residual macroscopic disease should be attempted in all advanced stage LGSOC patients. A meta-analysis of the German AGO (Arbeitsgemeinschaft Gynäkologische Onkologie) database involving 145 advanced stage LGSOC patients observed that a residual disease larger than 1 cm was associated with significantly impaired outcomes as compared to the patients without residual macroscopic disease (5-year overall survival of 32% (median 35 months, range 31–39 months) versus 85% (median 97 months, range 60–124 months; *p* < 0.001). Comparing no residual macroscopic disease to resection, with residuals up to 1 cm, revealed a significant death risk reduction of 55.3% (HR 0.447, 95% CI 0.297–0.673, *p* < 0.001) [4]. This observation was confirmed by both an Italian retrospective multicenter study and an ancillary study of the GOG182, analyzing 92 and 189 advanced LGSOC patients, respectively [25].

The role of systematic lymphadenectomy has long been debated. While the randomized controlled LION trial did not detect a survival benefit in favor of systematic lymphadenectomy but reported a significantly higher risk of postoperative complications, the study predominantly included patients with HGSOCs (n = 465 with HGSOCs versus n = 29 with LGSOCs) and subgroup analysis is not available [26]. A recent meta-analysis including 971 LGSOC patients with dominantly advanced stage disease also did not detect a survival difference following systematic lymphadenectomy (for OS: HR 1.15, 95% CI 0.42–3.18, I^2^ = 84%; and for PFS: HR 1.46, 95% CI 0.63–3.41, I^2^ = 71%) [27]. Both the ESGO–ESMO–ESP consensus conference recommendations and NCCN guidelines opt against routine systematic lymphadenectomy in ovarian cancer patients with FIGO stage IIIb or higher if no lymph node metastases are suspected [14,15].

### 2.2. Neoadjuvant Cytotoxic Chemotherapy Approaches

In contrast to HGSOCs, neoadjuvant chemotherapy (NACT) is not to be considered a standard approach in patients with advanced LGSOCs but may be an alternative in selected cases if patients are unfit for radical primary cytoreductive surgery or if residual disease < 1 cm cannot be achieved [14]. 

Several randomized clinical trials have evaluated the value of NACT for patients with advanced stage epithelial ovarian cancer, but no subgroup analysis for different histological subtypes is available to date [28,29,30,31]. Bonsang-Kitzis et al. demonstrated no significant survival difference between patients who underwent NACT followed by interval debulking surgery with residual disease of more than 0.25 cm and patients without any surgery. Moreover, NACT showed no benefit in terms of reduced surgical complexity in comparison to primary debulking surgery for patients with advanced LGSOCs. NACT followed by interval debulking surgery was linked to a higher Peritoneal Cancer Index (*p* = 0.03) and increased rates of digestive, diaphragmatic, and upper abdominal peritoneum involvement (*p* = 0.01; *p* = 0.003; *p* = 0.0001, respectively). Additionally, diaphragmatic peritoneum stripping (58.1% versus 36.7%) and bowel resection (54.8% versus 34.4%) were performed more frequently [32]. 

A retrospective analysis of 25 patients with an advanced LGSOC who underwent neoadjuvant platinum-based chemotherapy revealed an objective response rate of only 4% [33]. A meta-analysis using the National Cancer Database indicated that patients with an advanced LGSOC who were treated with neoadjuvant chemotherapy experienced worse OS compared to those who underwent primary debulking surgery (4-year rates, 56.4% versus 81.0%; HR 2.12; 95% CI, 1.55–2.90) [34].

A meta-analysis of the French multicenter ESME database, including 289 patients with advanced LGSOC, showed a significantly impaired OS in the groups which received NACT followed by interval debulking surgery or NACT +/− bevacizumab +/− maintenance endocrine therapy without surgery compared to the cohort which underwent primary debulking surgery (OS of 146.0 months for primary debulking surgery (95% CI, 110.4—not reached) versus 75.5 months (95% CI, 48.7—not reached) for NACT followed by interval debulking surgery versus 38.0 months (95% CI, 33.0–65.0) for NACT +/− Bevacizumab +/− maintenance endocrine therapy) [35]. Lorenzo et al. also associated NACT with an impaired OS (HR = 2.24, 95% Cl (1.08–4.66); *p* = 0.030) and identified NACT as an independent predictor of both impaired PFS (*p* = 0.010) and OS (*p* = 0.030) in advanced LGSOCs [25].

### 2.3. Adjuvant Cytotoxic Chemotherapy Following Primary Cytoreductive Surgery

According to the ESGO–ESMO–ESP and NCCN guidelines, the role of chemotherapy for FIGO stages IB–IC is uncertain and can be considered optional. If indicated, either six cycles of carboplatin monotherapy or a minimum of three cycles of carboplatin/paclitaxel should be applied in stage IB and six cycles of carboplatin/paclitaxel in stage IC [14]. According to the NCCN guidelines, no adjuvant chemotherapy is recommended in stage IB and three to six cycles of carboplatin/paclitaxel may be considered in stage IC [15]. Advanced stages (FIGO ≥ II) are recommended to undergo six cycles of adjuvant carboplatin/paclitaxel, according to both the ESGO–ESMO–ESP and NCCN guidelines [14,15]. 

Nevertheless, evidence has repeatedly confirmed the limited effectiveness of cytotoxic chemotherapy in LGSOCs [12,33,36]. A retrospective analysis of the AGO database confirmed a marked difference in response rates to platinum-based chemotherapy in advanced HGSOC (n = 218) and LGSOC patients (n = 145) with residual disease following primary cytoreductive surgery (90.1% versus 23.1%, *p* < 0.001). Of note, no significant survival difference between the two groups after primary cytoreductive surgery with residual disease of larger than 1 cm followed by platinum–taxane chemotherapy could be observed (5-year OS 10.9% versus 5.6%, *p* = 0.813; 5-year PFS 10.9% versus 5.6%, *p* = 0.813; respectively). This finding may be attributed to the higher responsiveness to chemotherapy in HGSOCs, which outbalances the lower proliferative activity and therefore longer time to progression in LGSOCs [4]. 

In line with this, a National Cancer Database analysis of 618 patients with stage II–IV LGSOC and residual disease after primary cytoreductive surgery did not observe a significant OS difference in the patients who received adjuvant chemotherapy (81.1%) as compared to the patients who did not (18.9%) (HR 0.87, 95%CI 0.55–1.39) after adjusting for stage and clinical confounders [37].

While evidence on carboplatin/paclitaxel as the most common adjuvant regimen is limited, efficacy data on alternative dosages or active agents are only anecdotic. Whether or not other strategies, e.g., involving cisplatin or dose-dense protocols, could be more effective than carboplatin/paclitaxel remains unknown. Despite the small numbers of LGSOC patient subgroups, neither the AGO-OVAR 3 (carboplatin/paclitaxel versus cisplatin/paclitaxel), the AGO-OVAR 5 (carboplatin/paclitaxel +/− epirubicin), the AGO-OVAR 7 (carboplatin/paclitaxel +/− topotecan), nor the AGO-OVAR 9 (carboplatin/paclitaxel +/− gemcitabine) reported marked differences in the outcome of specific regimens for LGSOC patients [38,39,40,41]. As the inherently lower mitotic activity of LGSOC does not appear to favor conventional cytotoxic therapy in general, and there is no common molecular aberration in LGSOC supporting the use of specific cytotoxic agents known to date (as, e.g., a therapeutic preferability of gemcitabine in case of ARID1A mutations in clear cell ovarian cancers has been hypothesized), optimizing the available adjuvant cytotoxic regimens does not appear to be a very promising strategy to improve therapeutic outcomes in the future [4,42]. 

#### 2.3.1. The Role of Bevacizumab during First-Line Therapy

The addition of bevacizumab to carboplatin/paclitaxel was approved by the EMA based on the ICON7 and GOG-0218 phase III trials, which assessed the potential therapeutic value of adding bevacizumab to chemotherapy in primary advanced ovarian cancer. In 2011, the ICON7 reported a significantly improved PFS in patients with high-risk or advanced ovarian cancers in the overall cohort (HR 0.81; CI 95%, 0.70–0.94; *p* = 0.004) [43]. Similarly, the GOG-0218 showed that the addition of bevacizumab during and up 10 months after platin-based chemotherapy was associated with prolonged PFS in patients with advanced ovarian cancers (HR 0.717; 95% CI, 0.625–0.824; *p* < 0.001) [44]. The final overall survival analysis of the ICON7 in 2015, however, failed to observe a statistically significant survival difference in the subgroup analysis of LGSOCs as compared to chemotherapy alone (mean survival time for 31 patients in bevacizumab arm of 50.4 months versus 50.5 months for 49 patients in the standard therapy arm) [45]. In contrast, the retrospective multicenter MITO22 study observed that adding bevacizumab (n = 30) to first-line chemotherapy appeared to improve the median PFS in advanced LGSOCs in comparison to platin-based chemotherapy alone (n = 65) (PFS 47.86 months (95%CI, 31.48—NR) versus 22.63 months (95%CI, 15–39.24), *p* = 0.0392) [46]. A recent systematic review evaluating the safety and efficacy of bevacizumab with or without cytotoxic chemotherapy in 153 LGSOC patients (adjuvant therapy: 80 patients of the ICON 7; recurrence therapy: 73, pooled data from 5 case series and retrospective cohort studies) estimated an overall response rate of 47.5%, which would be considerably higher than the previously reported results for the cytotoxic chemotherapy or hormonal therapy strategies. Of note, the reported response rates are only derived from studies assessing bevacizumab application in recurrent setting. The systematic review identified no LGSOC-specific new safety flags [47]. In summary, the additional therapeutic value of adding bevacizumab to first-line cytotoxic chemotherapy remains uncertain. While a subgroup analysis of the only prospective, randomized trial available to date yielded negative results, the ICON7 was neither powered nor designed to assess this research question in LGSOCs. Given the small number of patients at risk receiving bevacizumab, the absolute OS numbers appear to favor the study group, despite not reaching statistical significance. Smaller retrospective studies have reported seemingly more promising results. In line with the ESGO–ESMO-ESP and NCCN guidelines, bevacizumab can therefore be considered an additional therapeutic option in combination with adjuvant platinum-based chemotherapy in advanced LGSOCs [14,15]. As evidence remains limited, further clinical studies will be necessary to sharpen the role of bevacizumab during first-line therapy.

#### 2.3.2. The Role of Endocrine Therapy during First-Line Therapy

Advanced LGSOCs typically feature high expression levels of estrogen receptors (ER) in about 90% of patients and progesterone receptors (PR) in 40–50% of patients, providing a strong rationale for a potential therapeutic target [5,48]. Evidence on the efficacy of endocrine therapy during first-line therapy, however, remains limited to the following retrospective cohort study: Gerhenson et al. observed a significantly longer PFS in patients with LGSOC stages II–IV after primary cytoreductive surgery and platinum-based chemotherapy who received hormone maintenance therapy as compared to observation only (PFS of 64.9 months versus 26.4 months, *p* < 0.001) [24]. Patients with residual disease after the completion of primary therapy appeared to experience even greater clinical benefits from extra hormone maintenance therapy (38.1 months versus 15.2 months; *p* < 0.001). Extrapolating the data from a phase II trial on patients with recurrent LGSOCs, letrozole appears to be more effective than tamoxifen [49]. 

The following three prospective clinical trials are currently underway to finally define the role of endocrine therapy, holding the potential to reshape the therapeutic landscape of LGSOCs: The MATAO Phase III trial is currently underway to identify subgroups of epithelial ovarian cancer patients who may benefit from maintenance therapy with the aromatase inhibitor letrozole (NCT04111978). The trial assesses letrozole in 540 patients with newly diagnosed HGSOCs, LGSOCs, and endometrioid ovarian cancers with FIGO stages II–IV, who have undergone either primary or interval debulking surgery followed by at least four cycles of platinum-based chemotherapy. A key inclusion criterion is the presence of an estrogen receptor expression of 1% or greater [50]. The randomized, controlled NRG GY-019 phase III trial assesses the efficacy of adding letrozole to adjuvant carboplatin/paclitaxel chemotherapy followed by letrozole maintenance therapy in 450 patients LGSOC FIGO stages II–IV after primary cytoreductive surgery (NCT04095364) [51]. The ongoing randomized phase III LEPRE trial randomizes letrozole versus carboplatin/paclitaxel as adjuvant therapy following primary cytoreductive surgery in stages III–IV ER-positive LGSOCs (NCT05601700) [52]. 

Until results are available, endocrine therapy offers a cost-effective and readily available treatment option with promising efficacy. Both the ESGO-ESMO-ESP and NCCN guidelines discuss endocrine therapy maintenance after first-line platinum-based chemotherapy as a potential treatment option, although specific recommendations are not available, reflecting the limited evidence available to date [14,15]. 

## 3. Management of Recurrent Disease

Approximately 80% of patients with advanced LGSOCs experience disease recurrence and are subsequently referred to palliative therapy [11,53]. Given the modest response rates and significant side effects of platinum-based chemotherapy rechallenge, LGSOC recurrence poses a significant therapeutic dilemma [12]. Inherently low proliferation rates of LGOCs, however, often result in clinically slow-growing tumors, which can sometimes be controlled over a course of many years. Multiple therapy lines frequently lead to long-term toxicities, severely impacting the quality of life of affected patients [54]. As a result, both secondary cytoreductive surgery and targeted therapies are of increasing clinical interest. Endocrine, anti-VEGF, or anti-MAPK approaches may offer more personalized strategies to maintain patient quality of life and limit treatment toxicities. Evidence, however, remains limited and is primarily derived from early clinical trials and/or retrospective cohort studies. Table 2 summarizes clinical trials for recurrent LGSOCs with available results.

### 3.1. Secondary Cytoreductive Surgery

Expert panels encourage us to evaluate the option of secondary cytoreductive surgery if disease burden is limited and achieving no macroscopic residual disease is deemed technically feasible [14,59]. The randomized DESKTOP III trial demonstrated an OS benefit in 407 patients with recurrent platinum-sensitive ovarian cancer, with platinum-free interval of 6 months or more, undergoing secondary cytoreductive surgery followed by platinum-based chemotherapy (OS of 53.7 months (95%CI 46.8 to 61.6) versus 46.0 months (HR 0.75, 95%Cl 0.58–0.96), *p* = 0.02. However, only 10 patients with LGSOCs were included in the trial. In the overall cohort, patients achieving no macroscopic residual disease demonstrated a substantial prognostic benefit (OS of 61.9 months (95% CI, 55.3–78.9) versus 27.7 months (95% CI, 23.5–38.7)) [60]. In line with this, another retrospective study observed that complete resection in patients with relapsed LGSOC (n = 41) was associated with a significantly longer PFS compared to those residual disease (PFS of 60.3 months (95% CI, 0.0–123.9) versus 10.7 months (95% CI, 6.5–14.9), *p* = 0.0008) [61]. Secondary cytoreductive surgery in LGSOC (n = 29) patients was previously associated with higher surgical complexity (58.6% versus 36.8%; *p* = 0.05) including a higher risk of multiple bowel resections (24.1% versus 8%, *p* = 0.04) and higher median estimated blood loss (400 versus 200 mL; *p* = 0.01) in comparison to HGSOC patients (n = 87) [62]. Despite the absence of a sufficiently powered prospective trial involving a large number of LGSOC patients undergoing recurrence surgery, the lower sensitivity of LGSOCs to chemotherapy provides a compelling rationale to reconsider secondary cytoreductive surgery for these patients, when feasible, in line with both the ESGO–ESMO–ESP and NCCN guidelines [14,15].

### 3.2. Systemic Treatment Options for Recurrent Disease

Regardless of whether secondary cytoreductive surgery is performed, several systemic therapy options are available following disease recurrence. However, the evidence for these treatments is typically limited and mostly derived from retrospective studies or extrapolated from trials predominantly involving HGSOCs.

#### 3.2.1. Cytotoxic Chemotherapy

Cytotoxic chemotherapy reinduction as an effective mainstay treatment for recurrent HGSOCs is of very limited efficacy in recurrent LGSOCs, which have previously been labeled as “chemoresistant” in the literature. Gerhenson et al. reported an overall response rate of 4.9% for the “platinum-sensitive” (platinum-free interval of >6 months) cohort and 2.1% for the “platinum-resistant” (platinum-free interval of <6 months) cohort of 58 recurrent LGSOCs, who received 108 combinations of different chemotherapy regimens in the pre-bevacizumab era, whereas stable disease was observed in 60.2%. Of note, several different cytotoxic chemotherapy options, both with and without platinum, were recorded, and no specific regimen demonstrated a markedly better efficacy [12]. Outcomes following the physician’s choice of chemotherapy after ≥1 prior platinum-based chemotherapy but ≤3 prior chemotherapy lines were recorded as a 12.9% overall response rate, a PFS of 10.6 months, and an OS of 20.8 months in the MILO/ENGOT-ov11 [55]. The GOG-281/LOGS reports response rates to mono-chemotherapy in recurrent LGSOCs as 9% following paclitaxel mono (n = 1/11), 3% following pegylated liposomal doxorubicin (n = 1/40), and 0% following topotecan (n = 0/8) [49].

#### 3.2.2. Bevacizumab

The AURELIA trial, assessing the efficacy of combining bevacizumab with single-agent chemotherapy in recurrent ovarian cancer (weekly paclitaxel, pegylated liposomal doxorubicin, or topotecan), observed a significantly improved PFS in favor of the bevacizumab arm (PFS of 6.7 months versus 3.4 months, HR 0.48 (95% CI, 0.38 to 0.60); *p* < 0.001). However, out of the 361 patients included, only 19 (5.3%) had grade 1 tumors, and the study did not specify how many of these 19 tumors were of serous histotype, nor did it provide subgroup analyses on treatment efficacy according to the tumor histotype [63]. As early as 2014, a small exploratory retrospective study conducted at MD Anderson, Houston, TX, reported promising response rates of up to 55% if cytotoxic chemotherapy was combined with bevacizumab in patients with a recurrent LGSOC (n = 13) [64]. The MITO22 study observed a median PFS of 37.1 months (95%CI 13.42–40.56) versus 11.2 months (95%CI 8.26–15.63) in favor of adding bevacizumab to chemotherapy in 16 recurrent LGSOC patients, *p*-value = 0.013 [46]. A recent systematic review estimated an overall response rate of 47.5% to bevacizumab with or without cytotoxic chemotherapy by evaluating the pooled data of 73 patients from two case series and three retrospective cohort studies, not including the MITO22 [47].

#### 3.2.3. Endocrine Therapy

Endocrine therapy is a cornerstone in the management of recurrent LGSOC; Gershensen et al. retrospectively assessed 64 patients, reporting an overall response rate of 9% in a pooled analysis of several different therapeutic regimens [65,66]. The single-arm observational phase II PARAGON trial reported a response rate of 14% to anastrozole in a mixed cohort of 36 recurrent or metastatic ER-positive and/or PR-positive LGSOCs (n = 32), low-grade endometroid carcinomas (n = 2), and serous borderline tumors (n = 2). Even though no subgroup analysis of the LGSOC cohort is available, the results appear to be in line with the other available observational studies [66]. The prospective phase II/III GOG281/LOGS, which compared the efficacy of the MEK-inhibitor trametinib to other available therapeutic regimens including endocrine treatment, reported an overall response rate of 14% to letrozole (n = 44) and 0% to tamoxifen (n = 27) [49]. 

## 4. Novel Therapeutic Strategies and Future Directions

Given the modest response rates to cytotoxic chemotherapy, and only few therapeutic alternatives in case of disease recurrence, there is an urgent unmet clinical need to define novel therapeutic strategies to improve therapy outcomes and reduce the associated toxicities. Recent advances in understanding the molecular mechanisms underlying the pathogenesis and progression of LGSOCs have helped us to identify promising new therapeutic targets, particularly highlighting the efficacy of endocrine treatment and MAPK-blockade. Future directions in LGSOC treatments are likely to focus on clarifying the role of endocrine therapy in both the adjuvant and recurrent setting and on integrating approaches to block the MAPK pathways to overcome therapy resistance and achieve more durable clinical responses. Tumor genome sequencing and referral to a specialized oncologic center should therefore be considered for all the patients diagnosed with LGSOCs in the non-curative stages to facilitate the evaluation of novel targeted strategies for their potential enrolment in therapeutic trials. Table 3 summarizes the ongoing clinical trials for LGSOCs.

### 4.1. Endocrine Therapy

While results of the MATAO, NRG GY-019, and LEPRE trials are being eagerly awaited to finally define the role of the aromatase inhibitor letrozole during adjuvant therapy, elucidating the role of mutations in the ESR1 gene may help to further improve endocrine treatment efficacy. In ER-positive metastatic breast cancer, 30% to 40% of patients develop resistance to endocrine therapy over time, leading to clinical disease progression [73]. Mutations in the ESR1 gene, which encodes the ER, have been recognized as a major driving force behind this resistance and are now considered promising prognostic and predictive biomarker in this cancer type [74]. The literature on the role of ESR1 mutations in LGSOC patients is currently limited to the case reports describing the LGSOCs developing resistance to endocrine therapy associated with harboring ESR1 mutations [75,76]. Switching to selective ER modulators (SERMs) or selective ER downregulators (SERDs) after progression during AI treatment may offer a novel endocrine strategy [76]. The single-arm phase II FUCHSia basket trial recently evaluated the clinical efficacy of the SERD fulvestrant in recurrent gynecologic cancers, including four patients with LGSOC, all of whom achieved stable disease during fulvestrant treatment. Further clinical validation will be necessary to validate this therapeutic concept [67].

### 4.2. MAPK Blockade

The mitogen-activated protein kinase (MAPK) pathway is a critical signaling cascade regulating cell growth, proliferation, and survival [77]. Mutations in the genes encoding the components of the MAPK pathway, such as *KRAS*, *NRAS*, and *BRAF*, are frequently observed in LGSOCs, driving uncontrolled cell proliferation and tumor development [10,16,78,79]. MEK1/2, a central kinase within the MAPK pathway, is essential for transmitting the signals that promote tumor growth. Allosteric inhibitors of MEK1/2 activity have the potential to effectively disrupt downstream signaling, thereby inhibiting MAPK pathway signaling and deriving tumors of growth signals [80].

The MILO/ENGOT-ov11 phase III study evaluated the efficacy of the MEK inhibitor binimetinib in patients with recurrent LGSOCs against the physician’s choice of chemotherapy. The study closed early for crossing a prespecified futility boundary; binimetinib demonstrated a 16% overall response rate, 9.1 months PFS, and 25.3 months OS as compared to the 13% overall response rate, 10.6 months PFS, and 20.8 months OS following chemotherapy. Of note, *KRAS* mutations appeared to be linked to better therapy outcomes (NCT01849874) [55]. 

The single-arm phase II GOG-0239 trial evaluated the therapeutic efficacy of the MEK-inhibitor selumetinib in recurrent LGSOCs, reporting an overall response rate of 15.4% and a disease control rate of 63%. No association between the *RAS/RAF* mutational status and therapy outcomes was observed (NCT00551070) [56]. 

The randomized phase II/III GOG-281 trial compared the efficacy of the MEK inhibitor trametinib to established treatment options, in particular cytotoxic chemotherapy and endocrine treatments, observing a significantly improved PFS in favor of trametinib monotherapy at a response rate of 26% (overall cohort: PFS 13.0 months versus 7.2 months, HR 0.48 (95% CI, 0.36–0.64), unilateral *p* < 0.0001) (NCT02101788) [49]. These favorable results support trametinib as a promising new standard-of-care following disease recurrence.

Also, BRAF inhibitors, which have demonstrated efficacy in other cancers like melanoma, are being explored as potential therapeutic options in LGSOCs [81]. A recent phase I study of lifirafenib, a BRAF^V600E^ inhibitor, showed promising anti-tumor activity in a patient with solid tumors including LGSOC (NCT02610361) [82]. Furthermore, another BRAF inhibitor, ABM-1310, is currently being evaluated in a phase I trial enrolling patients with LGSOC harboring the *BRAF* ^V600E^ mutation (NCT04190628) [69]. 

Preclinical investigations demonstrate that dual RAF/MEK inhibition provides more prolonged pathway suppression compared to inhibiting RAF or MEK alone [83]. However, the compensatory activation of focal adhesion kinase (FAK) is believed to enhance the resistance to RAF/MEK pathway inhibition [84]. The combination of the FAK-inhibitor defactinib with the dual RAF/MEK inhibitor avutometinib (VS-6766) may overcome this resistance and has shown synergistic antitumor activity in *KRAS* wild-type LGSOC patient-derived tumor xenografts [85]. 

The combination of avutometinib (VS-6766) and defactinib demonstrated encouraging results in a phase I study with an overall response rate of 46% in recurrent LGSOCs (n = 24) (NCT03875820) [86]. Recently, an ENGOT-ov60/GOG-3052/RAMP-201 phase II study evaluating the activity of avutometinib (VS-6766) +/− defactinib presented preliminary findings, showing overall response rates of 7% (n = 2/30) for avutometinib (VS-6766) monotherapy and 28% (n = 8/29) for the combination of both (NCT04625270) [72]. Based on these promising results, the GOG-3097/ENGOT-ov81/RAMP 301 phase III randomized open-label study was initiated in March 2024 to evaluate the efficacy of the combination of avutometinib (VS-6766) with defactinib versus the investigator’s choice of treatment in recurrent LGSOCs (NCT06072781) [71].

### 4.3. CDK4/6 Blockade

Targeted sequencing often observes mutations in the CDKN2A gene (chromosome 9p) in LGSOCs; its gene product, the tumor suppressor p16, normally inhibits the G1 phase of the cell cycle via cyclin-dependent kinases (CDK) 4 and 6, which play a crucial role cell cycle progression and are responsible for phosphorylation of the retinobloblastoma (Rb) protein, inactivating its tumor suppressor function [87]. Deregulated CDK4/6 activity promotes uncontrolled cellular proliferation and is associated with the tumorigenesis of different types of cancer, including ovarian cancer [88,89]. Therefore, the presence of *CDKN2A* mutations may serve as a predictive biomarker for CDK4/6 inhibition in LGSOCs [90]. Early investigation with the CDK4/6 inhibitor palbociclib showed single-agent activity with limited efficacy in heavily pretreated unselected ovarian cancers (NCT01536743) [91]. A phase II trial combining ribociclib with letrozole demonstrated encouraging clinical activity in relapsed ER-positive ovarian (n = 20, thereof 3 LGSOC) and endometrial cancers (n = 20). The study observed promising response rates with a PFS of 12 weeks in 55% of patients; all three LGSOC were progression-free at a 24-month follow-up (NCT02657928) [92]. 

At ASCO 2023, the preliminary results of the GOG-3026 phase II trial reported an ORR of 24% (n = 9/37), with a clinical benefit rate of 86% following ribociclib and letrozole treatment in recurrent LGSOC patients (NCT03673124) [58]. Moreover, preliminary results from a phase II trial investigating neoadjuvant fulvestrant plus abemaciclib in advanced stage LGSOCs have reported a 47% overall response rate (n = 7/15), further supporting the potential of CD4/6 inhibitors (NCT03531645) [93]. While larger phase III trials are needed to confirm these initial findings, the use of CDK4/6 inhibitors in combination with other agents represents a promising future option for advanced LGSOCs.

### 4.4. Immunotherapy

Even though anti-PD-1/PD-L1 targeting revolutionized systemic therapy in several gynecologic cancers, genomic profiling does not provide a promising rationale for using checkpoint inhibitors in LGSOCs. In a cohort of 38 primary and 114 metastatic LGSOC tissue samples, ElNaggar et al. observed only a low prevalence of PDL1 expression (3%, n = 4), no case of microsatellite instability, and no case of a high tumor mutational burden [94]. In line with this, the phase II KEYNOTE-100 study assessed the efficacy of pembrolizumab monotherapy in patients with advanced recurrent ovarian cancer. A subgroup analysis of 21 (6%) patients with recurrent LGSOC revealed no case of an objective response [95]. Therefore, checkpoint inhibitor therapies should currently be limited to clinical trials. One ongoing phase II study (PERCEPTION/NOGGO-ov44) is currently investigating the potential efficacy of pembrolizumab in combination with chemotherapy in platinum-sensitive recurrent LGSOCs with platinum-free interval over 6 months and has recently shown promising early results (NCT04575961) [70]. 

### 4.5. Antibody–Drug Conjugates

Mirvetuximab soravtansine-gynx (MIRV), an antibody–drug conjugate (ADC) targeting folate receptor alpha (FRα), has emerged as a promising new therapeutic option for patients with recurrent platinum-resistant FRα-high OC. However, previous studies are limited to studies involving HGSOCs. The phase II SORAYA trial assessed the efficacy and safety of MIRV in patients with FRα-high, platinum-resistant, advanced HGSOC and demonstrated an overall response rate of 32.4% (95% CI, 23.6 to 42.2; *p* < 0.0001) at a tolerable safety profile (NCT04296890) [96]. The phase III MIRASOL trial randomized MIRV versus the physician’s choice of chemotherapy in platinum-resistant HGSOCs with high FRα tumor expression and reported improved survival in favor of MIRV (median OS of 16.5 versus 12.8 months, HR 0.67, 95% CI, 0.50 to 0.89), *p* = 0.005 (NCT04209855) [97]. Even though there are no clinical data on MIRV in LGSOC, Manning-Geist et al. reported FRα-high overexpression of 40% in LGSOCs. LGSOC tumors that lack alterations in the MAPK signaling pathway are more likely to exhibit FRα-high expression (61% versus 20%, *p* < 0.001) [98]. Therefore, FRα-directed ADCs warrant an investigation as a potential therapy option for LGSOC, particularly for MAPK non-altered tumors.

## 5. Conclusions

LGSOC is a rare subgroup of epithelial ovarian cancers for which the optimal treatment approach has yet to be established. Although the molecular biology and clinical behavior of LGSOCs differ from the more common HGSOCs, many treatment recommendations are still based on evidence from HGSOCs, as the low incidence of LGSOCs hampers both translational and clinical research. A growing body of evidence is questioning the role of cytotoxic chemotherapy in both primary and recurrent disease. The results of the MATAO, NRG GY-019, and LEPRE trials will be crucial in establishing a common adjuvant standard. Until then, platinum-based chemotherapy, with or without endocrine treatment and bevacizumab, can be considered for first-line therapy.

In cases of disease recurrence, patients should be carefully evaluated for secondary cytoreductive surgery, as platinum-based chemotherapy reinduction has shown low response rates. Depending on prior treatment lines, both endocrine therapy and bevacizumab remain viable therapeutic options.

Numerous promising targeted treatments are currently under evaluation, with clinical trials focusing on the CDK4/6 and MAPK pathways. The MEK inhibitor trametinib has shown promise in treating recurrent LGSOC and has been incorporated into the latest ESGO–ESMO–ESP and NCCN guidelines as a recommended option after platinum-based therapy failure.

Given its nature of being a rare disease and the lack of standardized treatment options, centralizing the oncologic treatment of LGSOC patients, particularly in cases of advanced and recurrent disease, ensures that affected patients receive optimal care and have access to the latest targeted treatment options and clinical trials.

## Figures and Tables

**Table 1:** Pathological characteristics of primary LGSOC and HGSOC.

	LGSOC	HGSOC
HR-expression:		
ER-positive	<90%	80–95%
PR-positive	40–50%	20–60%
Gene mutations reported:		
*TP53*	8%	>95%
MAPK-pathway:	46–58%	<12%
*BRAF*	8–13%	<1%
*KRAS*	21–33%	<12%
*NRAS*	8–22%	<1%
HRD	10.9% *	27%
*BRCA1*	8.7%	<15%
*BRCA2*	<1%	8%
Other HRD	2%	7%

LGSOC, low-grade serous ovarian cancer; HGSOC, high-grade serous ovarian; ER, estrogen receptor; PR, progesterone receptor; HRD, homologous recombination deficiency [6,7,8,9,10,16,17,18,19,20]. * According to Norquist et al. LGSOC had significantly lower mutation rate of *BRCA1* and *BRCA2*.

**Table 2:** Clinical trials for recurrent LGSOC treatment with prospective available results.

Study Name	Phase	Drug	Mode of Action	Target	Intervention	Number of Patients	ORR	Identifier
GOG 281/LOGS	II/III	Trametinib	MEK-inhibitor	MEK1/2	Standard-of-care group vs. Trametinib monotherapy	260	26% (24/130) 50% *RAS-*mut (11/22)	NCT02101788
		Letrozole	Aromatase inhibitor	ER			14%	
		Tamoxifen	SERM	ER			0%	
		Liposomal Doxorubicin	Anthracyclin				3%	
		Taxol weekly	Taxane				9%	
		Topotecan	Topoisomerase 1 inhibitor				0%	
MILO/ENGOT-ov11	III	Binimetinib	MEK-inhibitor	MEK1/2	Physician choice chemotherapy vs. Binimetinib monotherapy	303	16% (32/198) 44% *RAS*-mut (19/43)	NCT01849874
		Liposomal Doxorubicin	Antracyclin				14%	
		Taxol weekly	Taxane				15%	
		Topotecan	Topoisomerase 1 inhibitor				0%	
GOG-0239	II	Selumetinib	MEK-inhibitor	MEK1/2	Selumetinib monotherapy	52	15%	NCT00551070
ENGOT-GYN2/GOG-3051/BOUQUET	II	Cobimetinib	MEK-inhibitor	MEK1/2	Cobimetinib vs. Atezolizumab + Bevazicumab	23	25% *RAS/RAF-*mut and NF1 loss (2/8)	NCT04931342
GOG 3026	II	Ribociclib Letrozole	CDK4/6 inhibitor Aromatase inhibitor	CDK4/6 ER	Ribociclib plus Letrozole	37	24%	NCT03673124

ER, estrogen receptor; ORR, objective response rate; SERM selective estrogen receptor modulator [49,55,56,57,58].

**Table 3:** Ongoing clinical trials of novel therapies in LGSOC.

Study Name	Phase	Drug	Mode of Action	Target	Setting	Intervention	Identifier
NRG GY-019	III	Letrozole	Aromatase inhibitor	ER	adjuvant	Carboplatin/paclitaxel followed by maintenance letrozole vs. letrozole monotherapy	NCT04095364
ENGOT-ov54/MATAO	III	Letrozole	Aromatase inhibitor	ER	adjuvant	Carboplatin/paclitaxel followed by letrozole vs. followed by placebo	NCT04111978
LEPRE	III	Letrozole	Aromatase inhibitor	ER	adjuvant	Paclitaxel/carboplatin vs. letrozole monotherapy	NCT05601700
FUCHSia Study	II	Fulvestrant	SERD	ER	recurrent	Fulvestrant intramuscular	NCT03926936
ENGOT-ov70/ALEPRO	II	Abemaciclib Letrozoloe	CDK4/6 inhibitor Aromatase inhibitor	CDK4/6 ER	recurrent	Abemaciclib plus letrozoloe	NCT05872204
	I	ABM-1310	RAF inhibitor	BRAF	adjuvant	ABM-1310 orally	NCT04190628
PERCEPTION	II	Pembrolizumab	CPI	PD1-Rezeptor	recurrent	Carboplatin-based chemotherapy with pembrolizumab	NCT04575961
GOG-3097/ENGOT-ov81/ RAMP 301	III	Avutometinib (VS-6766) Defactinib	Dual RAF/MEK Inhibitor FAK Inhibitor	MEK1/2/RAF PTK2	recurrent	Avutometinib (VS-6766) plus defactinib vs. investigator’s choice of treatment	NCT06072781
ENGOT-ov60/ GOG-3052/ RAMP-201	II	Avutometinib (VS-6766)	Dual RAF/MEK Inhibitor	MEK1/2/RAF	recurrent	Avutometinib mono vs. in combination with defactinib	NCT04625270

ER, estrogen receptor; SERD, selective estrogen receptor degrader; CPI, checkpoint inhibitor; FAK; focal adhesion kinase [50,51,52,67,68,69,70,71,72].
